# Salivary Markers of Oxidative Stress and Their Relation to Periodontal and Dental Status Among Children With Down Syndrome

**DOI:** 10.7759/cureus.73852

**Published:** 2024-11-17

**Authors:** Nasreen H Hamonari

**Affiliations:** 1 Dental Public Health, Kurdistan Higher Council of Medical Specialities, Erbil, IRQ

**Keywords:** dmft index, down syndrome, malondialdehyde (mda), oral health, total oxidant status (tos)

## Abstract

Background: The current body of literature on oral health in individuals with Down syndrome (SD) presents contradictory information. Individuals with DS often have distinct oral health difficulties that set them apart from the general population.

Aim: This study aimed to measure malondialdehyde (MDA) and total oxidant status (TOS) levels in the saliva of children diagnosed with DS and explore their association with oral health status.

Materials and methods: A cross-sectional study enrolled 82 participants aged 7-12, comprising 40 children with DS and 42 controls matched based on age and gender. Diagnosing dental caries relied on the guidelines provided by the World Health Organization. Oral hygiene was examined with the plaque index, and gingival health was estimated using the gingival index (GI). To evaluate oxidative stress, unstimulated whole saliva samples were gathered to measure levels of MDA and TOS. The study data were analyzed using Statistical Package for the Social Sciences version 22 (IBM Corp., Armonk, NY). The p value for parametric variables was obtained using Pearson's chi-square test and Fisher's exact test.

Results: In this study, there were no significant differences between the two groups regarding periodontal health and dental decay. However, children with DS displayed significantly elevated GI values compared to the control group (p = 0.05). Children with DS demonstrated significantly higher MDA levels than the control group (p < 0.001). Furthermore, DS children demonstrated a higher prevalence of dental caries in their primary teeth than their permanent teeth.

Conclusion: Children with DS have comparable rates of dental caries to non-DS children but show elevated levels of GI values. Furthermore, children with DS exhibit high levels of saliva MDA compared to their peers without DS. Future research should include a wider age range, additional oxidative stress markers, and factors like diet, lifestyle, and antioxidant supplementation. Longitudinal studies and quality-of-life assessments could offer deeper insights into the oral health of individuals with DS.

## Introduction

Down syndrome (DS) is one of the most common genetic disorders globally, affecting approximately one in every 750-1,000 live births. It is characterized by the presence of an extra copy of chromosome 21, leading to various motor, behavioral, and physical challenges in those affected [1-4]. Individuals with DS have a higher prevalence of dental diseases than the general population. This increased susceptibility is often due to distinctive facial structures, altered muscle tone, and a higher incidence of medical conditions that affect oral health. Common oral health challenges in this group include dental cavities, gum disease, tooth loss, delayed exfoliation of primary teeth, supernumerary teeth, and dental misalignment [5,6]. Additionally, children with DS face significant difficulties in maintaining good oral hygiene due to cognitive impairments [7,8].

Oxidative stress occurs when the production of free radicals surpasses the body's antioxidant defenses, leading to cellular damage and increasing the risk of various diseases. It has been associated with developing oral conditions such as periodontitis and dental caries [9-11]. Studies have indicated that assessing oxidative stress markers in saliva can offer valuable insights into oral health conditions, as these markers are involved in oxidative pathways that contribute to the onset of periodontal disease and dental caries [10-14].

Assessing total antioxidant capacity (TAC) is crucial for quantifying oxidative stress, as it involves examining damage to lipids, proteins, and DNA. Malondialdehyde (MDA) is widely recognized as a key indicator of oxidative stress, primarily due to its role in lipid peroxidation [4,5,11-13]. Monitoring oral health within a population is vital for determining treatment priorities and devising effective preventive strategies. Addressing the specific needs of individuals with DS is essential for comprehensive healthcare planning [6,8,13].

Despite the extensive research on oral health conditions in individuals with DS, findings remain inconsistent, making it difficult to draw definitive conclusions. Additionally, limited research has explored the relationship between antioxidant systems and oral health in children with DS, underscoring the need for further investigation. The primary aim of this study was to evaluate and compare salivary oxidative stress levels between children with DS and their healthy siblings. The study also aimed to explore potential correlations between oxidative stress markers and the presence of dental caries and periodontal disease.

## Materials and methods

Study design and sample selection

A cross-sectional study was conducted between January and November 2023, involving 82 children aged 7-12 years from Erbil, Kurdistan Region, Iraq. Ethical approval was granted by the Scientific and Ethical Committee of the Kurdistan Higher Council of Medical Specialties on August 12, 2022 (reference number 2276).

Children with DS were recruited from the Helena Center, a government-run facility in Erbil specializing in services for children with special needs. The control group was drawn from Sabati Hawri, a nearby local state school, ensuring age and gender matching between the two groups. The inclusion criteria for the study were as follows: 1) a confirmed diagnosis of trisomy 21 (DS) from the Helena Center, 2) active cooperation from the children, and 3) parental or guardian consent. Exclusion criteria included children on long-term medication, those with severe systemic conditions or other disabilities, and children unable to comply with saliva collection procedures.

Before recruitment, the study's objectives were communicated to the administrative staff of the Helena Center and the selected school, and consent was obtained. Invitation letters containing consent forms were sent to parents, who were informed about the study's aims and data confidentiality. Participation was voluntary, and participants could withdraw at any time. A single examiner conducted all assessments to minimize bias.

Sample size determination

The sample size was calculated with an expected 50% prevalence of dental disease in children with Down syndrome, a 95% confidence level, and a margin of error of 10%. The initial required sample size was 96.04, which was increased by 20% to account for potential dropouts, resulting in a final target of 120 participants. The formula used for the calculation is as follows:

 n = Z^2^ * p * (1 - p) / e^2^

where n is the required sample size, z is the Z-score corresponding to the 95% confidence level (1.96), p is the expected prevalence of the condition (0.50), and e is the margin of error (0.10).

Recruitment and random sampling

Participants were selected using a simple random sampling method with a lottery system. Of the 152 eligible children with DS registered at the Helena Center, a government-run facility in Erbil for children with special needs, 60 were randomly selected to participate. Similarly, 60 children from the control group were also randomly selected from Sabati Hawri.

A total of 38 participants were excluded: 21 did not meet the inclusion criteria and 17 declined to participate. Following these exclusions, the final sample comprised 82 participants, including 40 children with DS (18 female and 22 male participants) and 42 children in the control group (22 female and 20 male participants). The distribution between the DS and control groups was well-balanced, which enhanced the validity of the analyses.

The total response rate for both groups was 68.33%. The Helena Center's registry facilitated accurate sampling, with staff contacting families, providing study details, and arranging appointments. For the control group, school staff contacted parents via phone calls and sent written notices requesting their consent and participation. No additional participants were excluded due to nonresponse or withdrawal.

Data collection and questionnaire

A proforma was created to collect detailed information about each child in the research and control groups. The proforma consisted of two sections: the first section involved conducting a comprehensive interview with the participants' parents to collect demographic information and inquire about their medical and dental backgrounds. The second section comprised a clinical examination to assess the presence of dental caries following the World Health Organization (WHO) [15] guidelines. Periodontal health was evaluated using the plaque index (PI) by Silness and Löe [16] and the gingival index (GI) by Löe and Silness [17]. Salivary oxidative stress markers were also measured. To ensure the clarity, reliability, and validity of the questionnaires, a pilot study was conducted by the researcher.

Dental caries were assessed by reporting the number of decaying (d, D), missing (m, M), and filled (f, F) teeth in both primary and permanent dentitions. The examination followed standardized protocols, and scores were designated decayed, missing, filled teeth (dmft) for primary teeth and Decayed, Missing, Filled Teeth (DMFT) for permanent teeth. The evaluation process, incorporating both tactile and visual criteria, followed a methodical technique, beginning with the final upper right tooth and progressing sequentially to the last lower right molar, reporting emphasized extensive and clinically evident carious surfaces per WHO guidelines [15]. For the assessment of periodontal status, the examination assessed four surfaces (buccal, lingual/palatal, mesial, and distal) of six index teeth (teeth 18, 23, 26, 38, 43, and 46) for dental PI and GI. Each site was probed with a periodontal probe, allowing 10 seconds to observe for gingival bleeding. Dental plaque was recorded if visible or if there was an accumulation of soft material in the gingival pocket or on the tooth and gingival margins, corresponding to scores 2 and 3 on the PI scale. Gingivitis was confirmed if bleeding occurred at any site, with scores 2 and 3 on the GI scale. Both indices ranged from 0 (excellent) to 3 (poor), with the GI assessing inflammation and the PI measuring plaque accumulation [14]. The composite scores for each participant were derived by combining both indexes. A comprehensive report outlining the oral health status of each child, their dental needs, and personalized recommendations for enhancing oral hygiene practices was provided to the parents.

Salivary sample collection

Unstimulated whole saliva was collected for five minutes between 8:00 am and 9:30 am to minimize any alterations due to circadian rhythm. The collection process was carried out with the assistance of the experienced staff at the Helena Center, who are well-versed in working with children with DS, ensuring a comfortable and safe environment for all participants.

Before sample collection, children were instructed to rinse their mouths with 15 mL of distilled water to eliminate food debris and exfoliated cells. Participants were then guided to assume the "coachman" position, with their heads slightly tilted downward, avoiding swallowing while allowing saliva to pool in the mouth. Saliva was collected into a clean graduated container until approximately 3 mL was obtained. The collected samples were immediately transferred to sterile Eppendorf tubes (Eppendorf Group, Hamburg, Germany), placed on ice at -4°C, and centrifuged at 3,000 rpm for 20 minutes to separate the various components of saliva, following procedures outlined in previous studies [7,10,14,18].

Some children were unable to provide saliva due to difficulty or lack of cooperation, so they were excluded. To avoid contamination from blood, intraoral assessments followed saliva collection.

Malondialdehyde assay

MDA assay measures MDA levels by reacting it with thiobarbituric acid (TBA) to produce a pink-colored complex, which is detectable at 535 nm. To prepare the MDA reagents, 0.375 g of TBA, 15 g of trichloroacetic acid (TCA), and 2.21 mL of 0.25 N HCl were combined and heated until fully dissolved, then cooled to 4°C. A standard MDA solution (16.4 μL) was diluted with distilled water to a final volume of 100 mL. This solution was mixed with saliva samples, and 1 mL of the TCA-TBA-HCl reagent was added. The resulting mixture was heated in a boiling water bath for 15 minutes. Following cooling and centrifugation, the supernatant was collected, and its absorbance was measured at 535 nm. MDA concentrations were calculated by comparing the absorbance to a standard reference curve, and results were expressed as µM/100 mL, with optical densities corrected for dilution factors [10,11,14].

Total oxidant status assay

Following Erel's 2005 method, the assay was employed to assess total oxidant levels in saliva [19]. The process began with mixing 225 μL of reagent 1, containing 150 μM xylenol orange, 140 mM NaCl, and 1.35 M glycerol in 25 mM H_2_SO_4_ (pH 1.75), with 35 μL of saliva. Absorbance was measured at 560 nm using a spectrophotometer with a sample blank. Next, 11 μL of reagent 2, which included 5 mM ferrous ion and 10 mM o-dianisidine in 25 mM H_2_SO_4_, was added. After a three-to-four-minute stirring period, the final absorbance was recorded at 560 nm. Total oxidant status (TOS) levels were determined by comparing absorbance values before and after the addition of reagent 2, with results expressed as μM H₂O₂ equivalent per liter (μmol H_2_O_2_ Equiv/L) [18].

Statistical analysis

The study data were analyzed using the Statistical Package for the Social Sciences version 22 (IBM Corp., Armonk, NY). The normality of the data distribution was assessed with the Kolmogorov-Smirnov test. Categorical variables were analyzed using the chi-square test and Fisher's exact test, while continuous variables were evaluated using the independent sample t-test and the Mann-Whitney test. Spearman's rho (ρ) was employed to assess correlations between MDA levels and oral health measures and between TOS and oral health measures in children with DS. A p value of ≤0.05 was considered statistically significant.

## Results

Approximately half of the sample consisted of male participants (55%), with no statistically significant difference in gender distribution between the two groups (p = 0.5). Additionally, as presented in Table 1, no significant differences in age were observed between the two groups (p = 0.2).

**Table 1:** Demographic characteristics of the study groups The chi-square test compares demographic characteristics between the Down syndrome and control groups ^*^p value of ≤0.05 was considered statistically significant

Variable	Study groups, n (%)		p value^*^
	Down syndrome	Controls	
Age			
<10 years	12 (30.0)	18 (42.9)	0.2
≥10 years	28 (70.0)	24 (57.1)	
Gender			
Male	22 (55.0)	20 (47.6)	0.5
Female	18 (45.0)	22 (52.4)	

The study found no statistically significant differences between the two groups in terms of DMFT (p = 0.3), dmft (p = 0.2), and PI (p = 0.9). However, there was a significantly higher mean GI among children with DS compared to controls (p = 0.05), as outlined in Table 2.

**Table 2:** Distribution of oral health measures according to study groups The Mann-Whitney test is used to compare nonnormally distributed variables, while the independent sample t-test is used for normally distributed continuous variables, assessing differences between the Down syndrome and control groups ^*^p value of ≤0.05 was considered statistically significant SD: standard deviation; DMFT: Decayed, Missing, Filled Teeth; dmft: decayed, missing, filled teeth; PI: plaque index; GI: gingival index

Oral measures	Study groups, mean ± SD		p value^*^
	Down syndrome	Controls	
DMFT	0.85 ± 1.12	1.09 ± 1.18	0.3
dmft	1 ± 1.1	1.35 ± 1.3	0.2
PI	0.67 ± 0.53	0.68 ± 0.6	0.9
GI	0.83 ± 0.69	0.57 ± 0.49	0.05

Children with DS had a notably higher mean level of MDA than the control group (p < 0.001). In contrast, no significant differences were observed between the two groups regarding salivary TOS (p = 0.08), as shown in Table 3.

**Table 3:** Distribution of salivary antioxidants measurements by study group Independent sample t-test used to compare normally distributed variables between groups ^*^p value of ≤0.05 was considered statistically significant SD: standard deviation; MDA: malondialdehyde; TOS: total oxidant status

Antioxidant measures	Study groups, mean ± SD		p value^*^
	Down syndrome	Controls	
MDA (µM)	134.9 ± 73.2	73.5 ± 44.28	<0.001
TOS (µM H₂O₂ equivalent)	26.4 ± 9.1	23.2 ± 7.89	0.08

As shown in Table 4, Spearman's rho coefficient reveals a significant positive correlation between MDA levels in children with DS and various oral health metrics: DMFT (p = 0.003), dmft (p = 0.01), PI (p = 0.009), and GI (p = 0.04).

**Table 4:** Spearman’s rho correlation of oral measures with MDA levels among children with Down syndrome Spearman’s rho (ρ) was used to assess the correlation between MDA levels and oral health metrics ^*^p value of ≤0.05 was considered statistically significant DMFT: Decayed, Missing, Filled Teeth; dmft: decayed, missing, filled teeth; PI: plaque index; GI: gingival index; MDA: malondialdehyde

Oral measures	Spearman's rho (ρ)	p value^*^
DMFT	0.46	0.003
Dmft	0.37	0.01
PI	0.40	0.009
GI	0.32	0.04

As indicated in Table 5, no significant correlation was found between the total oxidant status in children with DS and each of the following metrics: DMFT (p = 0.08), dmft (p = 0.52), PI (p = 0.8), and GI (p = 0.8).

**Table 5:** Spearman's rho correlation of oral measures with TOS among children with Down syndrome Spearman’s rho (ρ) was used to assess correlations between TOS and oral health measures in children with Down syndrome ^*^p value of ≤0.05 was considered statistically significant DMFT: Decayed, Missing, Filled Teeth; dmft: decayed, missing, filled teeth; PI: plaque index; GI: gingival index; TOS: total oxidant status

Oral measures	Spearman’s rho (ρ)	p value^*^
DMFT	-0.27	0.08
dmft	0.1	0.52
PI	-0.02	0.8
GI	0.3	0.8

No significant differences were found among children with DS across various age groups regarding DMFT (p = 0.8), PI (p = 0.5), GI (p = 0.7), MDA (p = 0.1), and TOS (p = 0.9). However, the dmft score was significantly higher among children with DS in the younger age group (p = 0.03) (Table 6).

**Table 6:** Distribution of study measures regarding children with Down syndrome age groups The Mann-Whitney test compared the dmft scores across age groups, while the independent sample t-test assessed differences in the DMFT index, PI, GI, and MDA levels, and TOS ^*^p value of ≤0.05 was considered statistically significant SD: standard deviation; DMFT: Decayed, Missing, Filled Teeth; dmft: decayed, missing, filled teeth; PI: plaque index; GI: gingival index; MDA: malondialdehyde; TOS: total oxidant status

Study measures	Age groups, mean ± SD		p value^*^
	<10 years	≥10 years	
DMFT	0.66 ± 0.77	92 ± 1.2	0.8
dmft	1.58 ± 0.99	0.75 ± 1.07	0.03
PI	0.75 ± 0.54	0.64 ± 0.53	0.5
GI	0.78 ± 0.55	0.85 ± 0.76	0.7
MDA (µM)	107.5 ± 51.6	146.6 ± 78.6	0.1
TOS (µM H₂O₂ equivalent)	26.4 ± 11.2	26.4 ± 8.2	0.9

As detailed in Table 7, no statistically significant differences were identified in the following parameters between male and female children with DS: DMFT (p = 0.7), dmft (p = 0.3), PI (p = 0.1), GI (p = 0.5), MDA (p = 0.6), and TOS (p = 0.7).

**Table 7:** Distribution of study measures concerning the gender of children with Down syndrome The Mann-Whitney test was used for nonparametric data. The independent sample t-test was applied to parametric data, including DMFT, malondialdehyde, and total oxidant status ^*^p value of ≤0.05 was considered statistically significant SD: standard deviation; DMFT: Decayed, Missing, Filled Teeth; dmft: decayed, missing, filled teeth; PI: plaque index; GI: gingival index; MDA: malondialdehyde; TOS: total oxidant status

Study measures	Gender, mean ± SD		p value^*^
	Male	Female	
DMFT	0.86 ± 1.08	0.83 ± 1.2	0.7
dmft	0.81 ± 0.95	1.22 ± 1.26	0.3
PI	0.57 ± 0.44	0.8 ± 0.6	0.1
GI	0.77 ± 0.7	0.91 ± 0.69	0.5
MDA (µM)	130.1 ± 75.8	140.7 ± 71.6	0.6
TOS (µM H_2_O_2_ equivalent)	26 ± 8.5	26.9 ± 10	0.7

## Discussion

Saliva is a valuable biofluid for clinical diagnostics, primarily due to its ease of collection, noninvasive nature, and minimal patient discomfort. This makes it an ideal medium for large-scale screenings, providing a cost-effective alternative to blood tests [4,5,7,9,12,20,21]. Salivary biomarkers, increasingly used to assess oxidative stress, play a critical role in understanding various oral health conditions in children [10,12,13].

In this study, unstimulated whole saliva was used, as it more accurately reflects antioxidant defense parameters and shows stronger correlations with clinical conditions than stimulated saliva [7]. Collecting stimulated saliva can be challenging in individuals with intellectual disabilities [5], making unstimulated saliva a more practical option for this population. Interestingly, no statistically significant difference was observed in the mean dental caries experience between the experimental and control groups, which is consistent with previous studies on individuals with DS [7,8,12,22-24]. While some research reports lower caries rates in children with DS [2,3,6,25-27], others find comparable or higher rates [7,8,12]. These inconsistencies could be due to factors such as biofilm formation and sugar consumption [12], as well as methodological limitations like small sample sizes and inconsistent participant ages.

This study aimed to evaluate the impact of DS on salivary factors, with a focus on periodontal health parameters, including the GI, probing depth, and bone loss. Gingivitis was the most prevalent periodontal issue, with significantly higher levels of gingival inflammation observed in the DS group, which aligns with previous studies [5,12,21,22,25,28,29]. Contributing factors to the elevated GI in children with DS include immunodeficiency [22,29], early colonization by periodontopathic bacteria [27,28], and difficulties in maintaining oral hygiene due to motor and cognitive impairments [5,7,8,21,29]. Faria Carrada et al. [21] observed that children with DS are more susceptible to periodontal changes, exhibiting a higher prevalence and density of specific pathogens that contribute to periodontal disease severity. Furthermore, oxidative stress has been proposed as a contributing factor to periodontal issues in this population [5,12,20].

In this study, the salivary TOS in children with DS was found to be comparable to that of healthy controls. This is consistent with findings by de Sousa et al. [4] and Zhang et al. [18], who reported similar TOS and antioxidant status in individuals with and without periodontitis or DS. De Sousa et al. [4] suggested that oxidative stress in saliva may be more closely linked to lipid peroxidation in individuals with DS. Additionally, elevated TAC levels may reflect an adaptive response to increased oxidative stress (TOS), as noted by Sánchez-Villamil et al. [30] and Su et al. [31]. The relationship between TOS and TAC highlights the body's efforts to maintain oxidative balance under stress, potentially mitigating oxidative damage in oral health.

A study by Anandan et al. [8] compared oral health parameters between children with DS (ages 5-18) and healthy controls, finding similar caries experiences and rates. The study also found that most salivary parameters did not significantly impact caries development in children with DS. Factors such as saliva collection methods, time of day, dietary antioxidant intake, and oral hygiene practices can influence salivary markers like TOS and TAC, which may help explain the complex relationship between oxidative stress and oral health in children with DS. Further research is needed to confirm these findings.

MDA, a marker for oxidative damage to biomolecules, was significantly higher in the saliva of children with DS compared to the control group, supporting the hypothesis of increased oxidative stress in this population [4,7,12,32]. Elevated MDA levels have also been observed in other tissues, such as plasma, brain, and lung, indicating systemic oxidative damage [4,7,12,30].

Although the overall prevalence of dental caries was similar between children with DS and the control group, the mean dmft (dmft in primary dentition) was significantly higher among younger children with DS. This finding aligns with Subramaniam et al. [7], who also reported a higher prevalence of caries in primary teeth than permanent teeth in children with and without DS.

Elevated MDA levels suggest a link between oxidative stress and oral health challenges in children with DS. Monitoring markers like MDA may provide valuable insights into oral health severity in this group. Future studies should explore antioxidant supplementation or other interventions to mitigate oxidative damage. Longitudinal research could refine clinical practices and improve preventive care. Expanding research to include adolescents and adults with DS will help identify age-related trends. Examining dietary and lifestyle factors may reveal modifiable influences. Combining longitudinal studies with quality-of-life assessments could further clarify the broader implications for this population.

Limitations and strengths

The study's primary limitation is that subjects with DS were selected from a specialized center, potentially limiting the generalizability of the findings. Recruitment challenges, particularly related to medication use and preexisting conditions, further contributed to this. However, strict adherence to inclusion and exclusion criteria minimized confounding factors, enhancing the reliability of the results. Additionally, the use of simple random sampling ensured a representative sample, strengthening the validity of the findings.

## Conclusions

The study revealed that children with DS exhibited a higher prevalence of dental caries in their deciduous teeth compared to non-DS children. However, the overall risk of dental decay in children with DS was similar to that of controls. Additionally, children with DS demonstrated poorer gingival health than their healthy counterparts. Both groups had comparable levels of salivary TOS, but the significantly elevated salivary MDA levels in children with DS support the hypothesis of increased oxidative stress in this population.
